# An *in vitro* evaluation of intravenous lipid emulsion on three common canine toxicants

**DOI:** 10.3389/fvets.2024.1482871

**Published:** 2024-09-25

**Authors:** Emery Jones, Stuart A. Walton, Jennifer Davis, McAlister Council-Troche

**Affiliations:** ^1^Desert Veterinary Medical Specialist, Phoenix, AZ, United States; ^2^Department of Small Animal Clinical Science, College of Veterinary Medicine, University of Florida, Gainesville, FL, United States; ^3^Department of Biomedical Sciences and Pathobiology, Virginia-Maryland College of Veterinary Medicine, Virgina Tech, Blacksburg, VA, United States

**Keywords:** intravenous lipid emulsion, baclofen, bromethalin, ibuprofen, toxicosis

## Abstract

**Objective:**

To determine whether intravenous lipid emulsion (ILE) therapy significantly reduces the concentration of baclofen, ibuprofen, and/or bromethalin in canine whole blood over time.

**Animals:**

Seven 500 mL bags of canine DEA 1.1 negative blood were divided into aliquots of 125 mL and randomly assigned to one of three treatment groups (baclofen, ibuprofen, bromethalin) or four control groups (a positive control for each treatment group and a negative control group).

**Procedures:**

Injectable ibuprofen (200 mg/kg), baclofen (8 mg/kg), or bromethalin (3 mg/kg) was apportioned into 125 mL aliquots of canine whole blood and incubated for 30 min at 38.5°C. ILE (12.4 mL, *Intralipid^®^*) was added to each sample and the solution vortexed [215 rpm for 15 min at 37°C (98.6°F)]. Samples were obtained at designated time points (0, 15, 30, 60, 180, 360 min), centrifuged, and separated into serum and RBC fractions. Serum samples were ultracentrifuged (22,000 *g* for 10 min at 37°C) to separate lipid rich and poor fractions. Samples were stored at −80°C prior to analysis.

**Results:**

A significant decrease in total drug concentration was established for bromethalin and its metabolite desmethylbromethalin compared to positive controls. ILE significantly reduced desmethylbromethalin at the 30-and 360-min time points. The remainder of the desmethylbromethalin time points did not reach significance. Bromethalin concentration was significantly reduced at all time points compared to positive controls. Neither baclofen nor ibuprofen had significant changes in concentration.

**Conclusion:**

ILE therapy was effective at reducing the total drug concentration of bromethalin and its metabolite desmethylbromethalin supporting the lipid sink theory. As a single compartment *in vitro* study, this study does not evaluate other proposed mechanisms of action of ILE therapy. ILE therapy may have other means of significantly decreasing lipophilic drug concentration in cases of toxicosis.

## 1 Introduction

In 2023, the ASPCA poison control center (APCC) received over 351,000 calls about potential pet poisonings (1). In the last 10 years, human medications and over-the-counter products have consistently topped the list of intoxications reported to the APCC (1, 2).

The use of intravenous lipid emulsion (ILE) infusion as an antidote in both human and animal toxicity cases has evolved over the last 20 years (3, 4). The first reported successful treatment of a lipophilic drug toxicity in veterinary literature occurred in 2009 when ILE was used to treat a puppy overdosed with moxidectin (3, 5, 6). Current veterinary literature on this topic is sparse and consists of review articles, case series, and case reports documenting the use of ILE in the treatment of a variety of lipophilic drug intoxications including baclofen, bromethalin, and non-steroidal anti-inflammatory drugs (NSAIDs) (3–13).

The mechanism of action of ILE in the treatment of lipophilic drug intoxication has not been fully elucidated (12, 13). It is likely that more than one mechanism of action contributes to the effects of ILE in cases of toxicity (4, 12, 13). The most popular theory is the “lipid shuttle” previously called the “lipid sink” theory (3, 4, 11–16). The “lipid sink” theory, first proposed by Weinberg and colleagues in 1998, theorized that intravenous lipid infusions created a lipid phase or compartment within the intravascular space, acting as a depot for lipophilic drugs to be partitioned into, resulting in the sequestration of potentially toxic substances away from vulnerable organs like the heart and brain (3, 4, 12, 13, 15–17). More recent, ongoing research supports a “lipid shuttle” theory. The lipid shuttle theory consists of two elements: (1) Lipid soluble drugs are drawn into the newly made lipid phase and are then (2) shuttled away from vital organs (heart and brain) and toward storage (adipose tissue and muscle) and detoxification organs (liver) (4, 14–16). An initial, transient increase in drug concentration, after initiation of ILE therapy has been reported in some patients (4). Improvement in clinical signs due to ILE therapy has been reported as soon as 30 min after initiation of ILE therapy (8). Other alternate theories for ILE’s mechanism of action include “mitochondrial recovery,” improved blood pressure support, and “direct inotropy” theories (3, 4, 12, 13). The “mitochondrial recovery” theory was first described with bupivacaine overdoses. The “mitochondrial recovery” theory suggest that ILE provides a source of energy to myocardial cells reducing bupivacaine’s cardiodepressant effects on cardiac myocytes (3, 12, 14, 18). ILE therapy improves blood pressure, possibly through vasoconstriction related to nitric oxide signaling or mediation of adrenergic sensitivity, thus improve outcome in intoxicated individuals (4, 14–16). In the “direct inotropy” theory, ILE has direct cardiac ionotropic effects through increased intracellular calcium concentration in cardiac myocytes (4, 14–16). The ionotropic effects of ILE lead to improved cardiac output (4, 14–16).

Lipophilicity of a drug, characterized by logP, evaluates the distribution between octanol and water (i.e., octanol–water partition coefficient) (4). Drugs with higher logP’s are more lipophilic and are expected to have a greater affinity to be cleared by ILE compared to drugs with a lower logP (12, 13). Drugs with a logP >1 are considered lipophilic (12, 13). Currently in veterinary literature there exist case reports and a single multicenter retrospective study detailing the successful clinical use of ILE in the treatment of baclofen, ibuprofen, and/or bromethalin intoxication (4, 5, 8, 9, 19, 20).

Baclofen (logP = −1.3), ibuprofen (logP = 3.97) and bromethalin (logP = 7.6) have varying degrees of lipophilicity (12, 13). The quantification of reduction of drug levels over time in patients treated with ILE for toxicity has yet to be evaluated.

Baclofen, a skeletal muscle relaxant, is prescribed for human patients with muscle spasticity and spinal disorders (21, 22). In dogs, baclofen has been intermittently used at a dose of 1–2 mg/kg once every 8 h in cases with urethral muscle spasticity (21, 22). While the toxic dose of baclofen has yet to be determined in dogs, the ASPCA reports lethal doses as low as 8-16 mg/kg (21). Common side effects in canines from baclofen intoxication include; ataxia, vomiting, vocalization, dyspnea, seizures, depression, hypothermia, paralysis of respiratory muscles, and hypotension (10, 21, 22).

Ibuprofen, a non-steroidal anti-inflammatory drug (NSAID), is commonly used for decreasing inflammation, analgesia, and as an antipyretic in human medicine (23–25). It is also one of the most common over-the-counter medications implicated in pet poisonings (23). Ibuprofen competitively and non-selectively inhibits both cyclooxygenase (COX) 1 and COX 2 (9, 23, 25). Toxicity is enhanced by enterohepatic recirculation, permitting continued re-exposure (9, 23, 25). Side effects of ibuprofen are dose dependent (9, 23, 24). Ingestion of ibuprofen doses ranging from 25 to 125 mg/kg may lead to vomiting, diarrhea, nausea, abdominal pain and anorexia. Ibuprofen doses >175 mg/kg have been demonstrated to lead to hematemesis, melena, and acute renal failure. While ibuprofen doses >400 mg/kg have been associated with seizures, coma, and shock (23).

Bromethalin, a dose dependent rodenticide, uncouples oxidative phosphorylation, resulting in reduced ATP production and disruption of ion channel pumps (26, 27). Canines ingesting large doses often develop hyperthermia, severe muscle tremors, hyperexcitability, focal and grand mal seizures, and death (27). Large doses include doses greater than or equal to the LD_50_ (3.65 mg/kg) (26). Small doses of bromethalin ingestion, those less than the LD_50_, are associated with slowly progressive symptoms which include hind limb ataxia and/or CNS depression (26, 27). Clinical signs and death have been reported in dogs after ingestion of bromethalin at doses as low as 0.46 mg/kg (27). Due to its wide availability and easy access, rodenticide poisoning is among the top ten reported toxicants in veterinary medicine each year (28).

While there are several studies in multiple species evaluating ILE for local anesthetic intoxications, most notably bupivacaine, there is no current data evaluating its ability to reduce drug concentrations of commonly encountered toxins in veterinary medicine (29–32). There are however multiple case reports in both human and veterinary literature that demonstrate the successful clinical use of ILE in the treatment of patients exposed to deadly doses of lipophilic drugs (5, 8, 11, 13). Given this shortcoming, there is a need to evaluate the utility of ILE for common lipophilic drug intoxications.

The purpose of this single compartment *in vitro* model is to evaluate the effect of Intralipid (20%) on the concentration of three common canine toxicants in whole blood over time and to calculate the percent reduction of these lipophilic drugs when ILE therapy is performed. The present study will be the first to evaluate the efficacy of ILE as an antidote for baclofen, ibuprofen, and bromethalin poisonings which are frequently encountered in veterinary medicine.

## 2 Materials and methods

### 2.1 *In vitro* susceptibility test

Seven 500 mL units of DEA 1.1 negative canine whole blood were purchased from a private blood bank (American Blood Resources International, ABR^®^). A blood gas was performed on a sample of blood obtained from each unit of blood. Each unit was equally divided into 4 aliquots of 125 mL each. Each of the original seven bags of blood were labeled with a letter A-G. Each aliquot was labeled with the letter from the parent bag (A-G) and a number (1–4), resulting in a unique identification for each aliquot. The aliquots were listed in an excel spread sheet column in letter and numerical order. In the column next to the aliquot identification numbers a random number was assigned to each aliquot using the function Rand(). The numbers and associated aliquots were then sorted from the lowest to highest random number. The aliquots were then assigned to one of 7 groups consisting of baclofen, ibuprofen, bromethalin, a positive control group for each of the three previous groups, and a single negative control group. Each treatment group was assigned 5 aliquots. The positive control group contained 2 aliquots per treatment group. The negative control group contained 2 aliquots total. The treatment group aliquots (baclofen, ibuprofen, bromethalin) were infused with the designated drug and ILE. The positive control aliqouts were infused with only the individual designated drug. The negative control aliquot was infused with ILE only. Each aliquot was equivalent to a 1.38 kg dog based on the assumption that a dog has 90 mL/kg of blood volume.

Aliquots were placed in a warm water bath at 37°C (98.6°F) for approximately 15 min. Aliquots were then moved to a temperature controlled orbital shaker set at 215 revolutions per minute (rpm) and 37°C (98.6°F) for 15 min to allow for equilibration. Each drug was instilled via needle and syringe into the designated aliquot. A total of 4 mg (3 mg/kg) of bromethalin, 11 mg (8 mg/kg) of baclofen, or 276 mg (200 mg/kg) of ibuprofen were added to individual aliquots. Each drug concentration was selected based on literature reported concentrations that have been associated with significant toxicity or mortality. The aliquot designated as the negative control, had no drug added to them. The aliquots were inverted by hand 10 times each and placed back in the temperature controlled orbital shaker at 215 rpm and 37°C (98.6°F) for 5 min.

### 2.2 Sample collection

Following equilibration and incubation, whole blood (3 mL) samples were aseptically collected Time (T0) via needle and syringe. The sample was directly placed into a 10 mL red top tube with no additives or separators. The tubes were centrifuged at 3,000 *g* for 10 min. After centrifugation the serum was removed and placed in a 2 mL microcentrifuge tube. The microcentrifuge tube was ultracentrifuged at 22,000 g and 37°C (98.6°F) for 10 min. After ultracentrifugation, the lipid rich and lipid poor samples were separated. All samples were stored in a temperature-controlled freezer set at −80°C (−112°F).

After collection of the T0 sample, 12.4 mL [1.5 mL/kg bolus +0.25 mL/kg/min (calculated for a 30-min period)] of intravenous lipid emulsion (ILE, Intralipid^®^20%) was added to each aliquot, except for aliquots designated as positive control. The aliquots were placed back on the temperature controlled orbital shaker at 215 rpm and 37°C (98.6°F). The previous process of removing, separating, and storing each sample was repeated at 15 min (min) after the infusion (T15), 30 min (T30), 60 min (T60), 180 min (T180), and 360 min (T360). Aliquots remained on the orbital shaker until the last sample (T360) was removed.

Two positive control samples were assigned via the random number generator to each drug group (baclofen, ibuprofen, bromethalin). The positive controls contained an equivalent amount of the designated drug as each study sample in their designated drug group. Each positive control was submitted to the same treatment and testing as the test samples without the addition of ILE. The positive controls were used to identify natural decay of each drug in the sample blood without the interference of intravenous lipid emulsion.

Two aliquots were randomly assigned to the negative control group. The negative control group contained an equivalent amount of ILE as the study samples. Besides for ILE, no other drugs were added to the negative control group samples. Aliquot handling and sampling were otherwise identical to the above outlined methods.

### 2.3 Sample analysis

Samples were analyzed at VetMed Analytical Research Laboratory at the Virginia-Maryland College of Veterinary Medicine Analytical Research Laboratory using techniques designed to evaluate the concentration of each individual drug. Ibuprofen concentrations were evaluated in both the lipid poor and the cellular portion of the samples as it is known to accumulate in red blood cells. All other drugs were measure in the lipid poor portion of the serum only. Ultra high performance liquid chromatography (UHPLC) with tandem mass spectrometry was used to measure both baclofen, ibuprofen, desmethylbromethalin concentrations were measured in the lipid poor portion of the serum. Bromethalin concentrations were determined via UHPLC with ultraviolet detection. All samples were run in duplicate. Appendix 1 provides a further detailed report of sample analysis.

### 2.4 Statistical and data analysis

A linear mixed model was used to model treatment effects over time, with the fixed effects being time point and treatment. To account for correlation in time caused by repeated measures an AR (1) correlation structure was included and variances were allowed to change with time. Linear contrasts were used to compare differences in the change from an initial time point, 15, between the treatment and the control. *p* values of non-independent contrasts were adjusted using the simulation method.

## 3 Results

Time 0 values were excluded from calculation due to at least one outlier (abnormally high drug concentration when compared to the rest of the samples) in each drug group at T0. These outliers were likely due to initial poor distribution of the drug in the sample canine blood bag. The pH of each parent bag of whole blood was found to range from 6.95 to 7.12.

### 3.1 Baclofen

The positive control had a mean total baclofen concentration of 118.3 μg/mL (SD +/−13.20) at T15. The lipid poor ILE samples had a mean total baclofen concentration of 109.3 μg/mL (SD +/−7.01) at T15. The reduction of baclofen concentration in the lipid poor ILE samples when compared to the control samples over time did not reach significance (*p* = 1.00).

**Figure 1:**
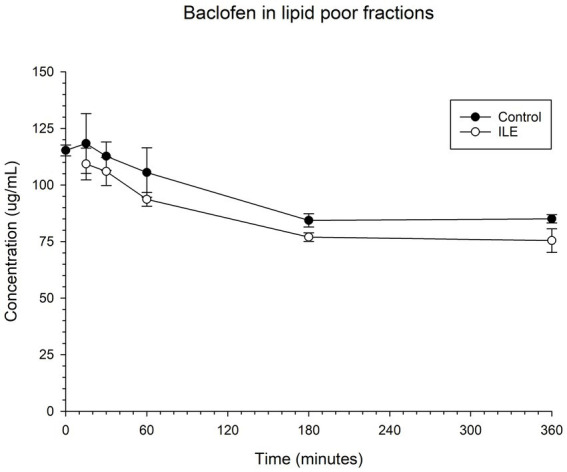
Compares the concentration of baclofen in the lipid poor fraction of the control and ILE baclofen treatment group over time.

The change in concentration of baclofen between the control samples and the ILE test samples did not reach significance with the inclusion of T0 with or without the exclusion of outliers at this time point. Table 1 and Figure 1 demonstrate the changes in the positive control and the lipid poor baclofen samples over time.

**Table 1:** Mean baclofen concentration and standard deviation (SD) at each time point for the lipid poor fractions of both the positive control and the ILE baclofen treatment group (ILE).

Time (minutes)	Control (μg/mL)	SD	ILE (μg/mL)	SD	% decrease
0	115.0	2.41			
15	118.0	13.20	109.0	7.01	7.7
30	112.0	6.30	106.0	6.21	6.0
60	105.0	10.9	93.7	3.01	11.3
180	84.40	2.94	77.0	1.87	8.8
360	85.06	1.82	75.4	5.20	11.3

### 3.2 Ibuprofen in plasma

**Figure 2:**
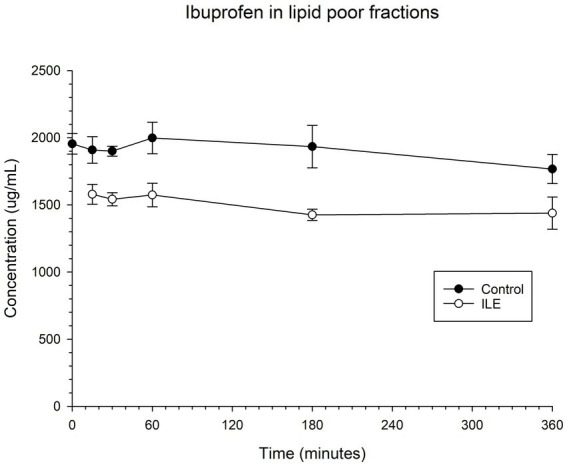
Compares the concentration of ibuprofen in plasma in the lipid poor fraction of the control and ILE ibuprofen treatment group over time.

The positive control had a mean concentration of ibuprofen of 1909.75 μg/mL (SD +/−98.99) at T15. The lipid poor ILE plasma samples had a mean concentration of ibuprofen of 1579.95 μg/mL (SD +/−74.12). The reduction in ibuprofen concentration in the lipid poor ILE plasma samples when compared to the control samples did not reach significance (*p* = 1.00) at any time point. Table 2 and Figure 2 demonstrate the changes in the positive control and Ibuprofen in lipid poor plasma samples over time.

**Table 2:** Mean ibuprofen in plasma concentration and standard deviation (SD) at each time point for the lipid poor fractions of both the positive control and the ILE ibuprofen treatment group (ILE).

Time (minutes)	Control (μg/mL)	SD	ILE (μg/mL)	SD	% decrease
0	1950.0	76.01			
15	1910.0	99.0	1580.0	74.1	17.3
30	1901.0	37.0	1540.0	49.0	18.9
60	2000.0	118.0	1570.0	87.5	21.2
180	1950.0	158.0	1430.0	41.7	26.3
360	1770.0	109.0	1440.0	120.0	18.6

The change in concentration of ibuprofen in plasma in the lipid poor ILE samples and control samples did not reach significance with the inclusion of time 0 with or without the exclusion of outliers.

### 3.3 Ibuprofen in red blood cells

**Figure 3:**
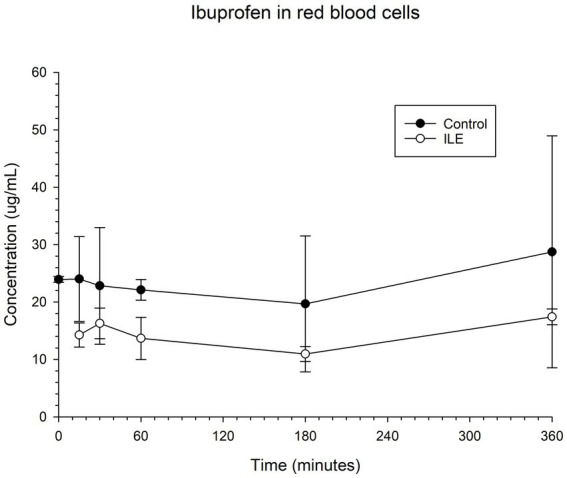
Compares the concentration of ibuprofen in red blood cells (RBC) in the lipid poor fraction of the control and ILE ibuprofen treatment group over time.

The red blood cells in the positive control had a mean concentration of ibuprofen of 24.00 μg/mL (SD +/−7.38) at T15. The red blood cells in the ILE samples had a mean concentration of ibuprofen of 14.25 μg/mL at T15. The total change in ibuprofen concentration in red blood cells in the ILE samples when compared to the control samples was not significant (*p* = 1.00) at any time point. Table 3 and Figure 3 demonstrate the changes in the positive control and ibuprofen in lipid poor plasma samples over time.

**Table 3:** Mean ibuprofen in red blood cells concentration and standard deviation (SD) at each time point for the lipid poor fractions of both the positive control and the ILE ibuprofen treatment group (ILE).

Time (minutes)	Control (μg/mL)	SD	ILE (μg/mL)	SD	% decrease
0	23.9	0.505			
15	24.01	7.38	14.2	2.11	40.7
30	22.8	10.2	16.3	2.66	28.6
60	22.1	1.80	13.7	3.68	38.2
180	19.7	11.9	11.0	1.30	44.3
360	28.7	20.2	17.4	1.38	39.4

The change in concentration of ibuprofen in red blood cells in the ILE samples and control samples did not reach significance with the inclusion of time 0 with or without the exclusion of outliers.

### 3.4 Bromethalin

**Figure 4:**
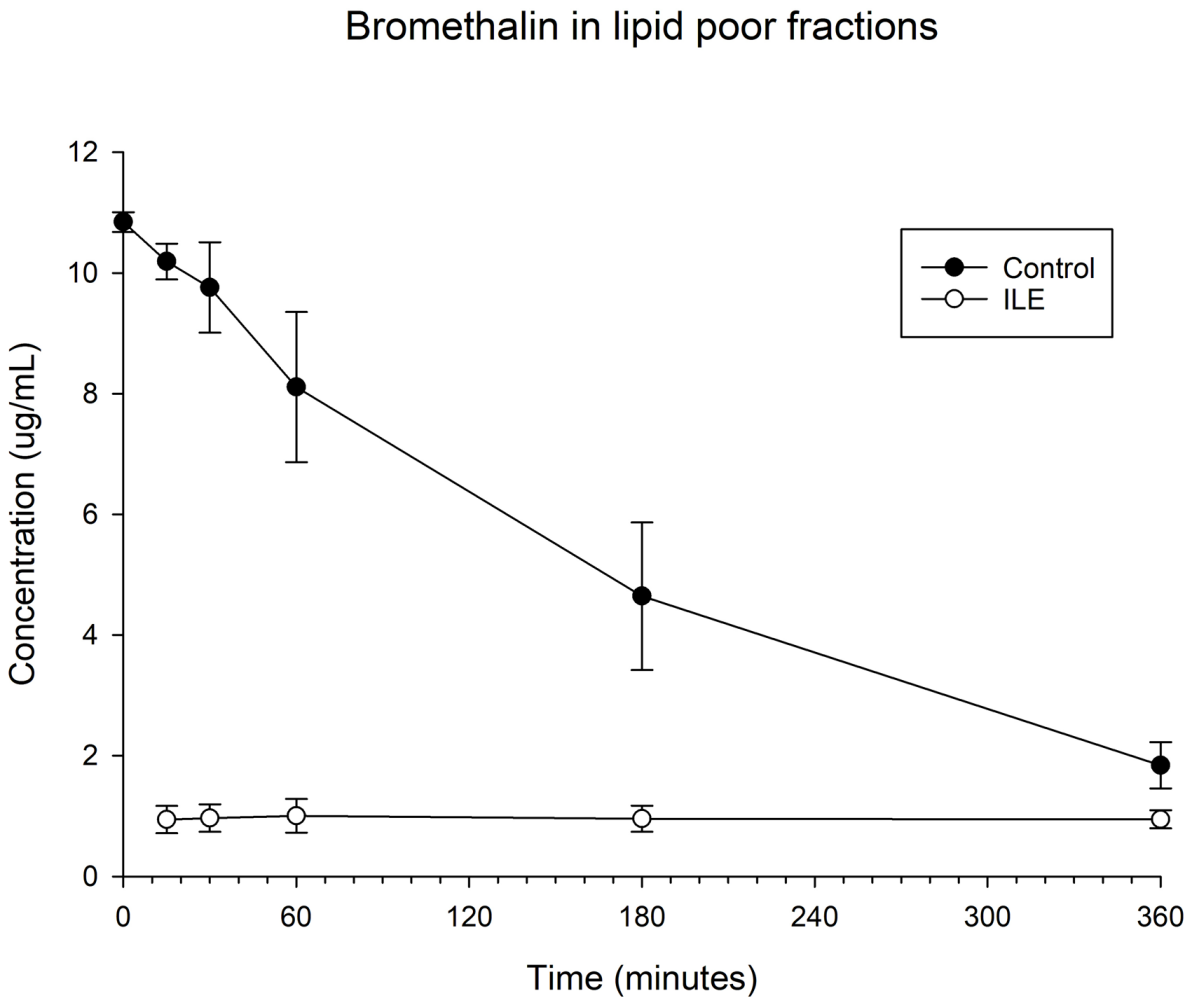
Compares the concentration of bromethalin in the lipid poor fraction of the control and ILE bromethalin treatment group over time.

The positive control samples had a mean total bromethalin concentration of 10.19 μg/mL (SD +/−0.30) at T15. The lipid poor ILE samples had a mean concentration of 0.93 μg/mL (SD 0.07) at T15. The bromethalin concentration in the lipid poor ILE samples was significantly different from the bromethalin concentration in the control samples at all time points (the highest *p* value = 0.05). Table 4 and Figure 4 demonstrate the changes in the positive control and lipid poor bromethalin samples over time.

**Table 4:** Mean bromethalin concentration and standard deviation (SD) at each time point for the lipid poor fractions of both the positive control and the ILE bromethalin treatment group (ILE).

Time (minutes)	Control (μg/mL)	SD	ILE (μg/mL)	SD	% decrease
0	10.8	0.16			
15	10.2	0.30	0.93	0.074	90.9
30	9.76	0.75	0.88	0.076	91.0
60	8.11	1.24	0.77	0.079	90.5
180	4.64	1.22	0.93	0.088	80.0
360	1.84	0.38	1.32	0.10	28.3

When values from time 0 were included, but the single outlier at time 0 is excluded, there was a significant difference in bromethalin concentration between the control sample and the ILE treatment sample at all time points except T360 (*p* = 0.19).

### 3.5 Desmethylbromethalin

**Figure 5:**
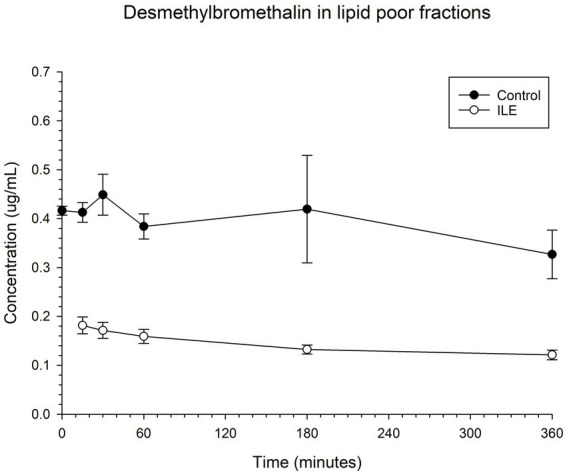
Compares the concentration of desmethylbromethalin in the lipid poor fraction of the control and ILE bromethalin treatment group over time.

The positive control had a mean concentration of desmethylbromethalin of 0.41 μg/mL (SD +/−0.02) at T15. The lipid poor ILE sample had a mean concentration of 0.18 μg/mL (SD+/−0.02), at T15. The reduction in desmethylbromethalin in the lipid poor ILE samples when compared to the control samples was significant at T30 (*p* = 0.01) and T360 (*p* = 0.02). All remaining time points did not reach significance. Table 5 and Figure 5 demonstrate the changes in the positive control and lipid poor desmethylbromethalin samples over time.

**Table 5:** Mean desmethylbromethalin concentration and standard deviation (SD) at each time point for the lipid poor fractions of both the positive control and the ILE bromethalin treatment group (ILE).

Time (minutes)	Control (μg/mL)	SD	ILE (μg/mL)	SD	% decrease
0	0.42	0.01	0.38		
15	0.41	0.02	0.18	0.02	56.1
30	0.45	0.04	0.17	0.02	61.8
60	0.38	0.03	0.16	0.01	58.6
180	0.42	0.11	0.13	0.01	68.5
360	0.33	0.05	0.12	0.01	62.9

When values from time 0 are included, but the single outlier at time 0 was excluded, there was a significant (*p* = 0.008) difference in the desmethylbromethalin concentration between the control sample and the ILE treatment sample at all time points except at the final time point T360.

## 4 Discussion

This single compartment *in vitro* study identified a significant decrease in the total drug concentration of bromethalin and its metabolite desmethylbromethalin after the administration of intravenous lipid emulsion (ILE). Bromethalin concentrations demonstrated a significant decrease at all time points. Reduction in desmethylbromethalin concentration after the administration of ILE was only noted to be significant at 30 (T30) and 360 (T360) minutes (6 h) after the addition of ILE. The remainder of the time points for desmethylbromethalin did not reach significance. A significant decrease in baclofen and ibuprofen concentrations after the addition of ILE was not identified.

Our study evaluated the scavenging potential of intravenous lipid emulsions in the “lipid shuttle” theory by determining the reduction in concentration of three drugs after the infusion of intravenous lipid emulsion in canine whole blood over time. The “lipid shuttle” theorizes that infused lipid creates a lipid phase or partition within the plasma. This newly created lipid phase draws in lipophilic drugs, shuttling these toxins away from target tissues (brain and heart) and toward tissues that store, metabolize, and/or excrete the toxin (13–16). The drugs evaluated in this study (baclofen, ibuprofen, and bromethalin) had varying levels of lipophilicity. Baclofen had the lowest logP at a logP of −1.3, which is below the threshold (LogP >1) for classification as a lipophilic drug (12, 13, 33). Due to baclofen’s low logP a lack of significant reduction in baclofen in this study is not surprising. Ibuprofen is considered a lipophilic drug with a logP of 3.97 (13). In the current study ibuprofen failed to demonstrate a significant decline in drug concentration with the administration of ILE. Based on the findings in this study, the previously reported improvement in patients treated with ILE for baclofen or ibuprofen toxicity is unlikely to be due to the theorized “lipid shuttle” but may be due to an alternate mechanism of action. Further studies are necessary to determine which, if any, previously proposed mechanisms of action of ILE therapy create the noted clinical improvement in patients with baclofen and ibuprofen toxicity. Bromethalin is the most lipophilic with the highest log *p* (7.6) of the drugs studied (13). The reduction of bromethalin after ILE infusion supports the “lipid shuttle” theory for this drug. Factors beyond LogP, like pH, may contribute to partitioning effects of ILE (15, 34). The pH dependent octanol–water partition coefficient, known as LogD, determines the lipophilicity of a drug at a certain pH (15, 34). Changes in pH can affect factors, like protein binding, altering the amount of free drug available to be scavenged by the lipid shuttle (34). Mazoit et al. demonstrated that by decreasing the pH from 7.40 to 7.00 the affinity of lipid emulsion to bupivacaine decreased by 1.68 fold (35). In a literature search evaluating each treatment drug information was lacking in regards to the log D of each drug in stored blood. Further research on the effects of pH on drug binding in lipid emulsions are necessary to further elucidate the effects of pH (15, 33, 34). ILE therapy would not be expected to alter the pH of blood in an alive animal and thus may not impact drug binding to the lipid emulsion in an *in vivo* model (36). Because LogD is dependent on pH it is often more difficult to measure (15, 34). The difficulty in measuring LogD due to the variation in pH has resulted in continued clinical use of LogP to determine lipoholicity and the likely success of treatment (15, 34).

Several case reports in human and veterinary medicine have reported a transient increase of lipophilic drug concentrations after initiation of ILE therapy, followed by a decrease in plasma drug concentrations (4, 37–39). Clarke et al. evaluated the response of a Border Collie with ivermectin toxicity to ILE therapy. Serum ivermectin concentrations were noted to increase shortly after a 1.5 mL/kg bolus of ILE was administered, but had decreased from baseline 30 min after initiation of the ILE constant rate infusion (CRI) (37). The increase in serum ivermectin levels post initiation of ILE was theorized to be due to sequestration of the toxin from tissues into the lipid phase, supporting the “lipid shuttle” theory (37). A significant increase in plasma drug concentration after ILE infusion followed by a decrease in plasma drug concentration was not found in this study as this model did not have a tissue compartment. Failure to demonstrate this phenomenon may support the theory that increases in plasma drug concentrations after the addition of ILE is related to the sequestration of drugs from tissue into the “lipid shuttle.”

Successful clinical recovery of humans and animals with reduction in plasma drug concentrations following ILE therapy has been published and the list of drug intoxications treated with ILE is ever expanding. This list includes local anesthetic agents (bupivacaine), amlodipine, amphetamines, baclofen, diltiazem, macrocyclic lactones (ivermectin, moxidectin), ibuprofen, permethrin, marijuana, phenobarbital, beta-blockers, carbamazepine, and bromethalin (3, 13, 15, 33). In our *in vitro* study, baclofen drug concentrations failed to demonstrate significant reduction with infusion of ILE therapy. Successful treatment of poorly lipophilic intoxicants like baclofen (LogP-1.32), is most likely related to other mechanisms of action like blood pressure support via alterations in nitric oxide signaling or direct cardiotonic effects (4, 13, 15, 16).

Our study found that the positive control for bromethalin demonstrated a steady decay in drug concentration over time. This is in contrast to the bromethalin treatment group following the addition of ILE, which demonstrated an almost immediate decrease in bromethalin concentrations. The steady decline of bromethalin in the positive control sample is likely due to natural decay and/or the development of breakdown products (40). Bromethalin has previously been noted to have a rapid photodegradation (27, 40). Lehner et al. found that bromethalin has the capacity to be degraded into up to 20 breakdown products (40). Unlike bromethalin, desmethylbromethalin has previously been found to undergo minimal photodegradation (27). While photodegradation and natural decay/conversion to desmethylbromethalin may have affected bromethalin concentrations, the significant decrease in bromethalin after introduction of ILE when compared to the positive control is supportive of the “lipid shuttle” mechanism of action.

Desmethylbromethalin, the active metabolite of bromethalin, was found to have a significant decrease in concentration when compared to the positive control at 30 and 360 min after ILE infusion. Desmethylbromethalin concentrations decreased at all time points when compared to the previous time point but failed to reach a significance at all time points. The presence of desmethylbromethalin in the samples evaluated in this study suggests that bromethalin was either converted to desmethylbromethalin in the blood bags or was present in the drug formulation infused into the blood bags. In the alive animal model, bromethalin is n-demethylated in the liver by cytochrome p450 to form desmethylbromethalin (27). Bromethalin and desmethylbromethalin reduce ATP production via uncoupling mitochondrial oxidative phosphorylation and disrupting ATP dependent sodium potassium ion channel pumps, thus leading to cerebral edema (27, 40). The authors speculate that in the alive model there would be a greater conversion of bromethalin to desmethylbromthalin due to hepatic metabolism, which may increase the binding and thus the beneficial effects of ILE therapy in the bromethalin intoxicated patient.

Serum concentrations of baclofen and ibuprofen did not significantly change after the addition of ILE when compared to the positive control. The historical successful use of ILE for baclofen and ibuprofen intoxications in veterinary medicine has been documented in several case reports and case series (5, 8–10, 41). Yet lack of improvement in toxin concentration in this study supports the existence of alternate mechanisms of action of ILE. Alternate proposed mechanisms of action include electrostatic interactions, blood pressure support via alterations in nitric oxide signaling, direct cardiotonic effects, and postconditioning effects that minimize reperfusion injury (15, 16, 34). If the successful treatment of these toxins is related to electrostatic interactions and thus LogD, as opposed to LogP, the relatively low pH of the stored whole blood could have impacted the lipophilicty of baclofen and ibuprofen, hindering the binding of the toxin to the lipid pool.

The free (plasma protein unbound) or unbound active drug is responsible for toxicity within the body. Of the drugs studied here, plasma protein binding of baclofen is low (approximately 30%) and ibuprofen protein binding is high (90–99%) (42, 43). Protein binding of bromethalin and desmthylbromethalin has not been reported. Based on this, baclofen free drug concentration would not be significantly affected by ILE therapy, while the lower than expected response of ibuprofen to ILE therapy may indicate a preference of the drug for binding to protein, rather than lipid. Further work is necessary to determine the free drug percentage of bromethalin and its metabolite in order to apply that knowledge to the results of this study.

Upon evaluation of the data sets for each drug it was noted that each drug had at least one sample with a significantly higher drug concentration than the other samples in the treatment group at time zero. None of the control samples were noted to have significant variation between individual samples at the same time point. Due to the presence of at least one outlier in each drug treatment group at time zero, the data for this time group was excluded from statistical analysis. Significant outliers were not noted at any other time point. The abnormally high drug concentrations in the time zero groups are believed to be due to poor mixing prior to sampling or continued drug residue in the sampling port.

ILE therapy was first suggested to be effective in the treatment of bupivacaine poisoning in 1998 by Weinberg et al. and was documented in the first human case report in 2006 (17, 44). Since the discovery of the benefits of ILE therapy for drug intoxication, ILE therapy has been used in both human and veterinary medicine for patients experiencing intoxication from both lipophilic and poorly lipophilic drugs (13, 15, 16, 32). Literature is dominated by case reports, case series, and review articles about the utility of ILE, the proposed mechanisms of action as well as the complications associated with the delivery of ILE (13, 15, 16, 34). However, research into mechanisms of action, appropriate treatment protocols, and possible adverse effects is still warranted. The mechanisms of action of ILE do not appear to be selective and target qualities many drugs and toxins share (4, 13). Due to the diverse proposed mechanisms of action, treatment with ILE could reverse crucial therapies used to stabilize patients experiencing a toxicity (4, 13). The potential reversal effect of these therapies should be considered prior to utilizing ILE therapy with drugs that are highly lipophilic (4, 13).

Based on case reports ILE therapy is widely accepted as a treatment for some drug intoxications and is typically well tolerated in the veterinary population (4). Recommended therapeutic protocols are extrapolated from human medicine (3, 4, 13, 32). General recommendations for ILE therapy in veterinary medicine is to use 20% lipid emulsion formulations and give a 1.5–4 mL/kg bolus over 1–15 min followed by a CRI of 0.25–0.5 mL/kg/min for 30–120 min (3, 4, 12). Pending response to this treatment, this protocol can be repeated. However, the total daily dose of ILE should not exceed 10 mL/kg/d (13).

Adverse effects associated with administration of ILE therapy in humans and animals include lipemia, pancreatitis, hemolysis, volume overload, facial pruritus, hypersensitivity reactions, infection associated with contamination of ILE product, and pain associated with extravasation of ILE (3, 4, 16, 34). Human patients intoxicated with drugs that have a large volume of distribution have been reported to experience a “rebound effect” after ILE therapy has been completed (4, 45). The “rebound effect” occurs when the lipid is cleared more quickly than a drug or toxin, resulting in a reoccurrence of clinical signs of toxicity (4, 45). In people ILE has also been associated with neurologic complications, acute kidney injury, acute respiratory distress syndrome, fat overload syndrome, and cardiac embolism (4, 16, 34). All patients receiving ILE therapy should be closely monitored until the gross lipemia has resolved (13, 34). If complications are noted during an ILE infusion the infusion should be discontinued.

All samples, except the positive controls, collected in this study at T15 and each time point after demonstrated evidence of gross lipemia. Gross hemolysis was present in all ibuprofen and bromethalin samples. Some level of hemolysis was present in all of the baclofen samples except the positive control and T0 samples. Lipemia was most significant in the negative control sample. ILE therapy has been reported to cause lipema and hemolysis in patients (4, 13, 34). Due to the presence of hemolysis in the positive control and the T0 samples, ILE therapy cannot be the only cause of hemolysis in the study performed here. Alternate possible causes of hemolysis include manual damage to red blood cells caused by ultracentrifugation or a reaction to the individual infused toxins.

### 4.1 Limitations

As a single compartment study this study did not evaluate other proposed mechanisms of action beyond the lipid shuttle theory. This study was also unable to evaluate the redistribution of toxin that has been proposed in the lipid shuttle theory. *In vivo* studies that compare findings to a control population could aid in further evaluation of the mechanisms of action, appropriate dosing regimen, and expected adverse effects. Due to the success reported in case reports and series, it is likely that alternate mechanisms of action play a role in the recovery of patients with intoxication of baclofen, ibuprofen, and bromethalin.

The findings of this single compartment *in vitro* study support the theorized “lipid shuttle” mechanism of action of ILE therapy in bromethalin intoxications. A significant decrease in baclofen and ibuprofen concentrations after the addition of ILE was not identified. Further studies are necessary to elucidate the benefits of ILE therapy for canine patients suffering from intoxication with baclofen and ibuprofen.

## Data Availability

The raw data supporting the conclusions of this article will be made available by the authors, without undue reservation.
