# Supplementary material for: An in vitro evaluation of intravenous lipid emulsion on three common canine toxicants
Source: Front Vet Sci. 2024 Sep 25;11:1482871. doi: 10.3389/fvets.2024.1482871 (PMC11461450; doi:10.3389/fvets.2024.1482871)
Supplement: Supplementary file 1 [file Data_Sheet_1.docx]

Appendix 1

**Ultra-High Performance Liquid Chromatography with Tandem Mass Spectrometry (UPLC-MS/MS) analysis of baclofen, ibuprofen, desmethylbromethalin and bromethalin in canine plasma**

Samples were extracted and analyzed at the VA-MD College of Veterinary Medicine Analytical Research Laboratory. Concentrations of baclofen (BAC), ibuprofen (IBU) and desmethylbromethalin (DMB) were determined by UPLC-MS/MS. Concentrations of bromethalin (BRO) were determined by HPLC-UV.

***Chemicals***

The BAC, IBU, and IBU-d3 reference standards were purchased from Cayman Chemical (Ann Arbor, MI, USA). The BAC-d4 internal standard was purchased from Sigma Aldrich (St. Louis, MO, USA). The BRO reference standard was purchased from LGC standards (Manchester, NH, USA). The DMB reference standard was purchased from Toronto Research Chemicals (North York, Ontario, Canada). Diphenylamine (DPA) was purchased from Fisher Scientific (Lenexa, KS, USA). Stock solutions of both all compounds were initially made up in their respective solvents and then separately diluted in acetonitrile (ACN) to their final standard concentrations.

***Sample Preparation***

All plasma samples were stored at -80 °C until prior to analysis and allowed to thaw at room temperature. All samples were subjected to protein precipitation followed by mixing, vortexing for 30 seconds and centrifugation at 16,100 x g for 10 minutes.

For BAC, plasma extracts were prepared by adding 180 µL of BAC-d4 (2 µg/mL in ACN+5%FA) to 20 µL of plasma in 0.6 mL microcentrifuge tubes. Supernatants were then diluted 1:200 in 10/90/1 ACN/H_2_O/formic acid (FA) for analysis. For IBU, plasma extracts were first diluted 1:50 by adding 20 µL of plasma to 980 µL deionized water, then 20 µL of the diluted plasma was then added to 180 µL of IBU-d3 (2 µg/mL in ACN) in 0.6 mL microcentrifuge tubes. Supernatants were then diluted 1:50 in 10 mM ammonium acetate (NH_4_Ac) in 10/90 ACN/H_2_O for analysis. For DMB, plasma extracts were prepared by adding 300 µL of DPA (133 ng/mL in ACN) to 100 µL of plasma in 0.6 mL microcentrifuge tubes. Supernatants were then diluted 1:10 in 50/50 ACN/H_2_O for analysis. Bromethalin was then determined from the same extracts by diluting the supernatant 1:10 in ACN.

Determination of IBU concentrations in RBC was performed on a mass basis followed by washing twice with 0.9% (w/v) NaCl solution to remove any excess plasma on the RBC samples. To the cell pellet, 50 µL of IBU-d3 (50 µg/mL in ACN), along with 950 µL of ACN were added before briefly vortexing and shaking then sonicating for 30 minutes to break up and extract the drug from the RBC. These were then centrifuged and diluted in the same manner as the IBU in plasma.

***Chromatography***

Sample extracts for BAC, IBU and DMB were subjected to chromatographic separation on a Waters H-Class UPLC system (Waters Corporation, Milford, MA, USA) using a phenyl column (Waters Acquity UPLC BEH Phenyl, 100 mm length x 2.1 mm ID x 1.7 µm) and matching guard column (Waters Acquity UPLC BEH Phenyl VanGuard Pre-Column, 5 mm length x 2.1 mm ID x 1.7 µm) maintained at 40°C. Five microliters of sample were injected onto the column using a refrigerated autosampler maintained at 8 °C.

For baclofen, the mobile phase consisted of solution A, 1% (v/v) formic acid (FA) in H_2_O, and solution B, 1% (v/v) FA in ACN. For IBU, the mobile phase consisted of solution A, 10 mM NH4Ac in H2O, and solution B, 10 mM NH4Ac in 90/10 ACN/H2O. For DMB, the mobile phase consisted of solution A, 0.1% (v/v) FA in H2O, and solution B, 0.1% (v/v) FA in ACN. The mobile phase was delivered to the UPLC column at a flow rate of 0.4 mL per min for each analyte. The gradient elution program is shown in Table 1A for BAC and DMB. The gradient elution program for IBU is shown in Table 1B.

For BRO, sample extracts were subjected to chromatographic separation performed on an Agilent 1100 HPLC with two Poroshell columns (Agilent EC-C18, 50mm length x 2.1 mm ID x 2.7 µm particle size) in tandem along with a matching guard column. Ten microliters of sample was injected onto the column using a refrigerated autosampler maintained at 8 °C. Mobile phase A was 100% H_2_O and mobile phase B was 100% ACN. The gradient used a flow rate of 1 mL/min and was similar to the one shown in Table 1A. UV detection and quantification of BRO was performed at 350 nm.

**Table 1A.** UPLC gradient method used for the chromatographic separation of BAC, DMB, and BRO.

| **Time (mins)** | **%A** | **%B** |
| --- | --- | --- |
| 0.00 | 50 | 50 |
| 0.25 | 50 | 50 |
| 2.75 | 2 | 98 |
| 3.25 | 2 | 98 |
| 3.26 | 50 | 50 |
| 5.25 | 50 | 50 |

**Table 1B.** UPLC gradient method used for the chromatographic separation of IBU.

| **Time (mins)** | **%A** | **%B** |
| --- | --- | --- |
| 0.00 | 75 | 25 |
| 0.25 | 75 | 25 |
| 2.50 | 2 | 98 |
| 3.00 | 2 | 98 |
| 3.01 | 75 | 25 |
| 5.00 | 75 | 25 |

Detection of BAC, IBU, and DMB was performed on a triple-quadrupole mass spectrometer (Waters Xevo TQD, Waters Corporation, Milford, MA) equipped with a Zspray ionization source (ESI). This was operated in ESI+ mode for BAC, ESI- for IBU, and operated simultaneously in ESI+/- modes for DMB. Multiple reaction monitoring (MRM) events were used to quantify the analytes of interest and their respective parent and product ion transitions are shown in Table 2.

**Table 2**. MRM transitions and specific mass spectrometry tuning parameters for the quantification of analytes.

| **Analyte** | **Parent Ion**  **(amu)** | **Product Ion**  **(amu)** | **Cone Energy**  **(V)** | **Collision Energy (eV)** | **Quant/Qual Transition** |
| --- | --- | --- | --- | --- | --- |
| BAC | 214.0  [M+H]^+^ | 150.9 | 24 | 18 | Quantifier |
|  | 214.0  [M+H]^+^ | 115.6 | 24 | 32 | Qualifier 1 |
| BAC-d4 (IS) | 218.1  [M+H]^+^ | 119.15 | 26 | 28 | Quantifier |
|  | 218.1  [M+H]^+^ | 154.5 | 26 | 20 | Qualifier 1 |
| DMB | 561.8  [M-H]^-^ | 278.1 | 52 | 30 | Quantifier |
|  | 561.8  [M-H]^-^ | 254.1 | 52 | 25 | Qualifier 1 |
|  | 561.8  [M-H]^-^ | 452.9 | 52 | 25 | Qualifier 2 |
| DPA (IS) | 170.1  [M+H]^+^ | 92.9 | 40 | 24 | Quantifier |
|  | 170.1  [M+H]^+^ | 65.0 | 40 | 30 | Qualifier 1 |
|  | 170.1  [M+H]^+^ | 152.2 | 40 | 24 | Qualifier 2 |
| IBU | 205.0  [M-H]^-^ | 161.0 | 18 | 8 | Quantifier |
| IBU-d3 (IS) | 208.1  [M-H]^-^ | 164.1 | 18 | 6 | Quantifier |

Commercial software (MassLynx) was used to analyze the data. Tuning was performed on each analyte by direct infusion of standard solution (0.1 ng/µL) at a rate of 10 µL per min. Mass spectrometer parameters used for detection are shown in Table 3.

**Table 3.** Mass spectrometer tuning parameters for the analysis of BAC, DMB and IBU.

| **Parameter / Analyte** | **BAC (ESI+)** | **DMB (ESI-)** | **IBU (ESI-)** |
| --- | --- | --- | --- |
| Capillary (kV) | +1.30 | -0.50 | -0.50 |
| Cone (V) | 24 | 55 | 18 |
| RF (V) | 2.50 | 2.50 | 2.50 |
| Extractor (V) | 3.00 | 3.00 | 3.00 |
| Source Temperature (°C) | 150 | 150 | 150 |
| Desolvation Temperature (°C) | 600 | 600 | 500 |
| Cone Gas Flow (L/Hr) | 10 | 10 | 10 |
| Desolvation Gas Flow (L/Hr) | 1000 | 750 | 1000 |

***Method Validation***

Our laboratory uses previously published guidelines for method validation from the USP-NF. Standard curves are made fresh for each analyte so that all concentrations back calculate to within ± 15% of the intended value and the curves are linear over the concentration range tested with an R^2^ >0.99. Validation data for each analyte in this study is shown in Table 4. The limit of detection (LOD) was based off of a signal to noise ratio of 3. The limit of quantification was based off of the lowest concentration shown to be linear on the standard curve.

**Table 4.** Recorded LOD, LOQ, range of standards concentrations used for the calibration range and the coefficient of variation for each analyte and matrix.

| **Analyte** | **LOD** | **LOQ** | **Calibration Range** | **CV** |
| --- | --- | --- | --- | --- |
| BAC | 0.08 ppm | 0.72 ppm | 0.72 – 180 ppm | < ± 10% |
| BRO | 0.1 ppm | 0.30 ppm | 0.30 – 30 ppm | < ± 10% |
| DMB | 0.6 ppb | 0.06 ppm | 0.06 – 1.95 ppm | < ± 10% |
| IBU (plasma) | 5.0 ppm | 50 ppm | 50 – 4,050 ppm | < ± 10% |
| IBU (RBC) | 0.19 ppm | 0.58 ppm | 0.58 – 230 ppm | < ± 10% |
